# Piebald Camels

**DOI:** 10.1186/s13570-017-0075-3

**Published:** 2017-02-20

**Authors:** Gabriele Volpato, Maurizio Dioli, Antonello Di Nardo

**Affiliations:** 1 Center for Integrative Conservation Research, University of Georgia, Athens, GA USA; 2 Independent Veterinarian, Alfaz del Pi, Spain; 3 The Pirbright Institute, Pirbright, Woking, Surrey UK

**Keywords:** Livestock breeds, Leucism, Paint dromedary camels, Pastoral nomads, Sahara

## Abstract

Animal breeds are the diverse outcome of the thousands-year-long process of livestock domestication. Many of these breeds are piebald, resulting from the artificial selection by pastoralists of animals bearing a genetic condition known as leucism, and selected for their productive, behavioural, or aesthetical traits. Piebald dromedary camels have not been studied or discussed before, and their same existence is often overlooked. Based on fieldwork in Western Sahara, direct observations across Northern and East Africa and the Middle East, and a literature review, we address the morphological and behavioural traits, geographical distribution, taxonomy, and material and cultural importance of piebald (painted) camels. They are a hundreds-year-old camel breed used for caravans, as mounts, and for aesthetical and cultural reasons across Sudan, Niger, Mali, Mauritania, Western Sahara, and Morocco. While they are increasingly bred out of a pastoral context for tourism and entertainment in the Canary Islands, mainland Europe, and the USA, in part of their original African range, piebald camels are under threat due to wars, droughts, and demise of pastoral livelihoods. More research is needed about these ‘beautiful and dignified’ animals.

*Have you ever seen a piebald* mahri^1^ *before?;*

*Have you ever seen a camel with such grace, lightness, and stature?*

*Have you ever seen anything more beautiful and dignified?*

(Al-Koni 2008)

## Introduction

A breed is a group of domesticates that has specific characteristics or traits artificially selected by man and transmitted through inheritance (Driscoll et al. 2009; Larson and Fuller 2014). Breeds are the diverse outcome of the thousands-year-long process of livestock domestication (Francis 2015). They have been selected in accordance to productive, cultural, and aesthetical traits and are often a key element of pastoral livelihoods and identities. Breed diversity is nowadays endangered by processes of livestock intensification and cultural homogenization (Faye et al. 2004), and as such, its study is a fundamental step for breeds’ conservation. Among the thousands of breeds from tens of domesticates existing in the world, several are piebald, i.e. spotted, painted, or patched of white and solid colour. Many of these piebald breeds are the result of a selective breeding for a genetic condition known as leucism.

Animals’ colouring is the result of either presence or absence of the pigment melanin in the skin, hair, and eyes. Among the known conditions affecting melanin production and animal colouring are albinism, melanism, and leucism; the latter resulting from defects in pigment cell differentiation and/or migration from the neural crest to the skin, hair, or feathers during development (Cieslak et al. 2011; Wilkins et al. 2014). In its most common form, leucism results in irregular patches on the body surface expressing as white on an animal that otherwise has normal colouring and patterning (Rook et al. 1998; Cieslak et al. 2011); when this happens, the animal is ‘pied’ or ‘piebald’ (also ‘paint’, ‘spotted’, or ‘speckled’).^2^ Some leucistic animals also exhibit coloration of the irises that matches the surrounding skin (blue eyes for pink skin, brown for dark).

Leucism and piebald colorations occur sporadically in the wild, as they usually reduce the individuals’ selective fitness providing less colour protection from predators (Woolf 1991). But recessive conditions in domesticates have been propagated voluntarily or not by humans since the very beginning of the domestication process, and changes in size and colour are among the first effects (Epstein 1955; Trut et al. 2009; Ludwig et al. 2009). Voluntary propagation includes the selective breeding of specific phenotypes for their religious, ritual, social, or subsistence/economic value. Involuntary propagation relates to the genetic and physiological links existing between tameness (a goal of early domesticators) and colour phenotypes (Wilkins et al. 2014; Cieslak et al. 2011; Price 2002) and to the effects of genetic drift in domesticates’ isolated populations (Zeder 2012). There is a general agreement that a wide variation of colour phenotypes were brought about by domestication, ‘but it is still largely unknown how color phenotypes were distributed in domesticated animals in earlier times, which roles they played for humans during these times and when they first occurred’ (Cieslak et al. 2011).

The piebald leucistic phenotype is known in a variety of domesticates, including Tobiano and Appaloosa horses (Brooks and Bailey 2005; Brooks et al. 2007), Belgian Blue and Shorthorn cattle breeds (Seitz et al. 1999), goats, and sheep, as well as dogs, cats, and pigs (Cooper et al. 2005; Giuffra et al. 1999). Several piebald animals have evolved into valued breeds, where Holstein cows and Dalmatian dogs are among the most notable examples.

Some of the genetic mutations related to the piebald character bear pleiotropic effects such as deafness, developmental disorders of the eyes and night blindness, and osteopetrosis (Stritzel et al. 2009; Bellone 2010; Wilkins et al. 2014). Because the development of the optical system is highly dependent on the presence of melanin, depigmentation has an effect on the development of the visual system (Grandin and Deesing 1998; Wilkins et al. 2014). Congenital stationary night blindness has been reported in a variety of spotted and piebald animals (e.g. Appaloosa horses; Sandmeyer et al. 2007). Animal breeders throughout the world have since early times recognized that a lack of body and eye pigmentation may be accompanied by neurological defects. A relationship between depigmentation and congenital deafness has been found, among other domesticates (Webb and Cullen 2010), in dogs (e.g. Dalmatian dogs with extensive white depigmented areas are most likely to be deaf; Strain 1996) and in llamas and alpacas (Camelids), where it is associated with pale blue eyes (Gauly et al. 2005).

A great deal of research has been conducted on the genetics, behavioural, and productive aspects of piebald breeds among several species (e.g. horses, dogs). In spite of this, the same existence of piebald camels has been largely overlooked, even in the scientific literature on camels (Wardeh 2004). Little and scattered information is available on piebald (painted) dromedary camels, and to the best of our knowledge, no single article has ever been written about them. The only author that has addressed their existence is Bulliet (1975), while too often it has not been properly acknowledged. For example, in the most recent and otherwise comprehensive book on animal domestication (Francis 2015), while discussing the correlation between coat colour and camel domestication, the author states that ‘In the United States, paint camels – white and brown, like pinto horses – have been produced, but paint camels are virtually absent in Arabia and North Africa.’ Though it is true that there are no painted camels in Arabia and East Africa, we contend that they indeed exist in North Africa since hundreds of years, and that the USA is just the latest country where they have been introduced after their diffusion westward from central-eastern Sahara, where they probably originated.

This paper departs from and contributes to the study of human-animal relationships and domestication from an historical and anthropological perspective (Bulliet 2005; Francis 2015; Hurn 2012). Most research on camels revolves around their potential as milk and meat producers and associated conditions (e.g. diseases) (Farah and Fischer 2004), whereas relatively less studies have been carried out on the productive and cultural links between pastoral populations and camels. In general, camel breeds are not as differentiated and classified as in other livestock species and their classifications are often derived from names of ethnic groups or geographical breeding regions rather than based on phenotypic characters (Dioli 2016). Therefore, camel breed study could help their conservation and support camel pastoralists (Kakar et al. 2011). Studies on the cultural relevance of piebald livestock (e.g. cattle) have already proven to be useful in describing complex human-animal relationships particularly in regard to livestock management (e.g. in genetic selection) and cultural identity (e.g. founding myths, cultural identity, and social values) (Coote 1994). In this paper, we address piebald camels’ physiological, genetic, and behavioural characteristics; their geographical distribution; taxonomy; and material and cultural roles among pastoral populations. Then, we discuss piebald camels’ origin and diffusion through Africa and out of Africa, we address trends in piebald camels’ husbandry, and we invite further research about and support to these ‘beautiful and dignified’ animals.

## Study area

**Figure 1:**
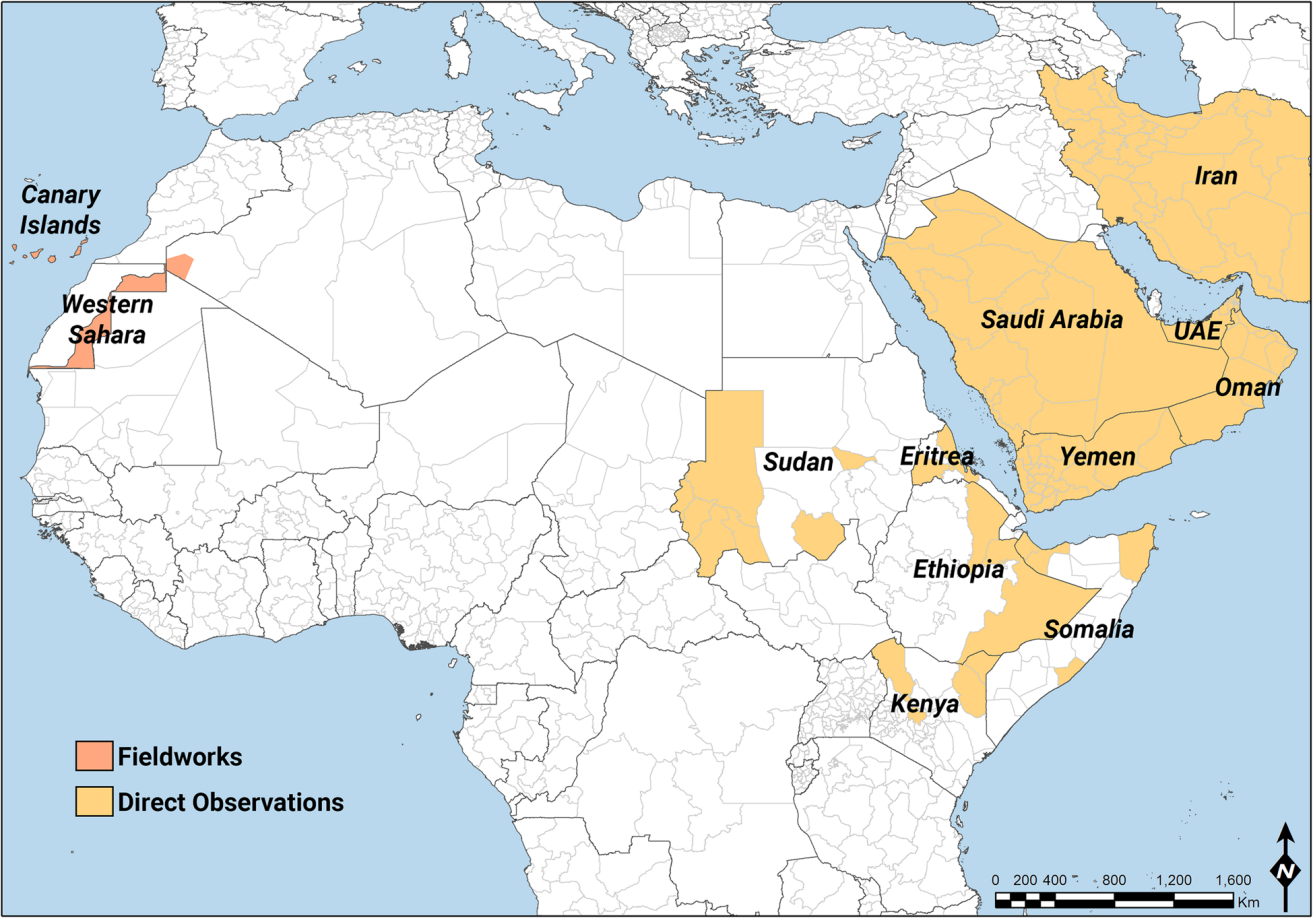
Map of the areas where fieldwork and direct observations have been conducted

The area under study includes large parts of the Sahara desert where camels are bred, with a focus on Western Sahara and the Sahrawi pastoralists (Figure. 1, Table 1). The Sahara is bordered by the Atlantic Ocean on the western edge, the Atlas Mountains and the Mediterranean Sea to the north, the Red Sea on the east, and the Sudan and the valley of the Niger River on the south. Half of the Sahara receives less than 25 mm of rain per year, while the rest receives up to 100 mm per year. Most of the Sahara is characterized as rocky hamada, a type of desert landscape defined by barren rocky plateaus. Large areas are covered by sand and dunes. The central part has extremely limited vegetation, while the northern, southern, and western reaches of the desert, and the highlands and mountain areas (e.g. Aïr, Hoggar, Saharan Atlas, Tibesti Mountains), have sparse grassland and desert shrub, with trees (mainly *Acacia* species) usually along the dry riverbeds (Julivert 2003). Across most of the Sahara, the dromedary camel (*Camelus dromedarius* L.) is the main livestock species. It was progressively introduced to large areas of the Sahara beginning about 2,500 years ago, and it provides pastoralists with milk, meat, and transport, as well as with a means of utilization of the local desert environment (Gauthier-Pilters and Dagg 1981).

**Table 1:** Location, duration, and time periods of fieldwork and direct observations

Time period	Duration	Country	Region	Type of study
1981 to 1991	10 years	Kenya	North west North east	Direct observations
1992 to 2013	3 years	Somalia	Middle Shebelle (Jowhar) Awdal (Borama) Bari (Bosaso) Woqooyi Galbeed (Berbera) Somaliland Puntland	Direct observations
1998 to 2001	3 years	Ethiopia	Afar Dire Dawa Somali (Jijiga, Ogaden)	Direct observations
2003 to 2006	3 years	Eritrea	Anseba (Keren) Gash-Barka (Barentu, Tesseney) Northern Red Sea (Nakfa, Massawa)	Direct observations
2004	Weeks	Western Sahara		Fieldwork
2006	Weeks	Kingdom of Saudi Arabia		Direct observations
2006 to 2007	Weeks	Canary Islands	Fuerteventura Lanzarote	Direct observations
2007 to 2009	1 year	Sudan	Darfur Khartoum (Omdurman) South Kurdufan (Nuba)	Direct observations
2008 to 2010	Months	Western Sahara		Fieldwork
2009 to 2010	3 months	Yemen		Direct observations
2010	Weeks	Iran		Direct observations
2010 to 2011	Months	United Arab Emirates		Direct observations
2012	2 weeks	Oman		Direct observations
2012	Months	Kenya	North west North east	Direct observations
2016	Weeks	Kingdom of Saudi Arabia		Direct observations

In-depth fieldwork was conducted in inland Western Sahara, northern Mauritania, and the part of Algeria to the south and south-east of the Hamada of Tindouf, which are the customary nomadic territories of Sahrawi pastoralists. Across this area, the climate is continental: summer daytime temperatures pass 50 °C, while winter night-time temperatures may drop to 0 °C. Rainfalls are torrential, unpredictable, and patchy, with an average annual rainfall of 30 to 50 mm and recurrent droughts. Generally occurring from the end of the summer through autumn, these rains are driven by the extreme northerly penetration of the African Monsoon from the south or are associated with the Atlantic Westerlies (Brooks et al. 2005). Biogeographically, we can distinguish two main areas: Zemmur to the north and Tiris to the south. The first runs east-west between northern Western Sahara and northern Mauritania: it is characterized by gravel plains with occasional surface of sandstone and granite in its eastern and central parts and by higher relief and hilly terrain in its western part. All Zemmur, and especially its central and western areas, is drained by inactive or occasionally active river channels that flow west into the Saguia el-Hamra, a large ephemeral river. After the rains, Zemmur displays a savannah-like environment dominated by Acacia-Panicum vegetation, while flowering prairies may appear on flat gravel areas. The southern sector, known as Tiris, is more arid and characterized by flat sand and gravel plains from which characteristic black granite hills arise in either clusters or in isolation. In Tiris, there are no dry riverbeds, and hence, vegetation is mostly herbaceous and adventitious and includes large areas covered by halophytic plants (Soler et al. 1999). For a background on the Sahrawi refugees and nomads and on their camel husbandry, see Volpato and Howard (2014) and Caro Baroja (1955).

## Methods

The data analysed in this paper are drawn from three types of sources: (i) a review of scientific and grey literature about piebald camels and related topics, (ii) direct observations, and (iii) fieldwork carried out between 2008 and 2010 in the Sahrawi refugee camps of west Algeria and in Western Sahara.

Fieldwork included semi-structured interviews (*n* = 32) with Sahrawi camel owners (26 of them had piebald camels in their herds) about morphological, physiological, and behavioural characteristics of piebald camels and their uses and cultural values. Interviews were conducted in Hassaniya (the Arabic language with Berber substrate spoken by the Sahrawi), recorded and translated into Spanish by local research assistants. Interviews were recorded and transcribed with the help of the same research assistant to minimize translation errors and clarify information. Transcripts were then entered into NVivo qualitative data management software, and codes, concepts, and categories were generated during analysis. In every case, prior informed consent was obtained verbally before the interview was conducted, according to the ethical guidelines adopted by the American Anthropological Association (2009) and by the International Society of Ethnobiology (2006).

Besides fieldwork in Algeria and Western Sahara, direct observations on the presence (or absence) and distribution of piebald camels have been conducted by the authors in the Canary Islands (2006 to 2007), Sudan (2007 to 2009), Kenya (2012, 2015), Ethiopia (1998 to 2001), Eritrea (2003 to 2006), Somalia (2013), Yemen (2009 to 2010), Oman (2012), UAE (2010 to 2011), Kingdom of Saudi Arabia (2006), and Iran (2010) (Table 1). The areas where fieldwork and direct observations have been conducted are represented in Figure 1 with a focus on Africa, where piebald camels are found. While fieldwork included interviews about piebald camels, direct observations are reports from the authors on piebald camels’ presence in the areas where they have worked or conducted research and did not include interviews about piebald camels.

## Results and discussion

### Morphological, physiological, and behavioural characteristics

**Figure 2:**
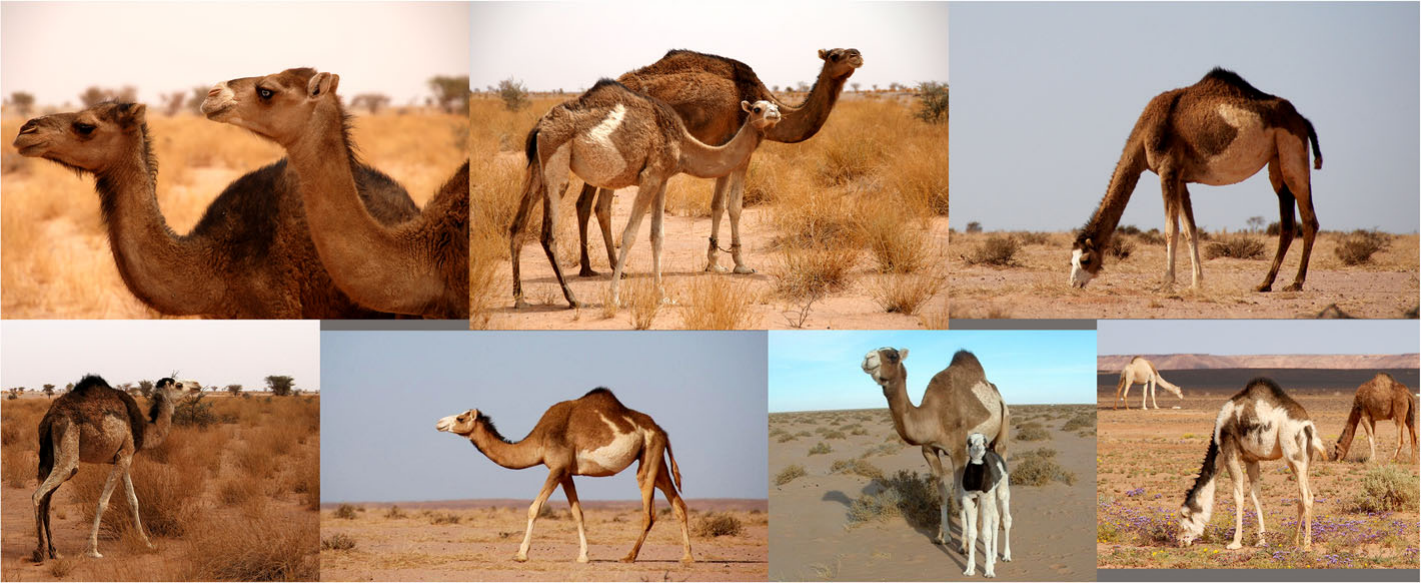
Table of pictures of piebald camels from Western Sahara showing the different colour patterns present in piebald camels and the white colour’s progression under distinct degrees of leucism (GV)

**Figure 3:**
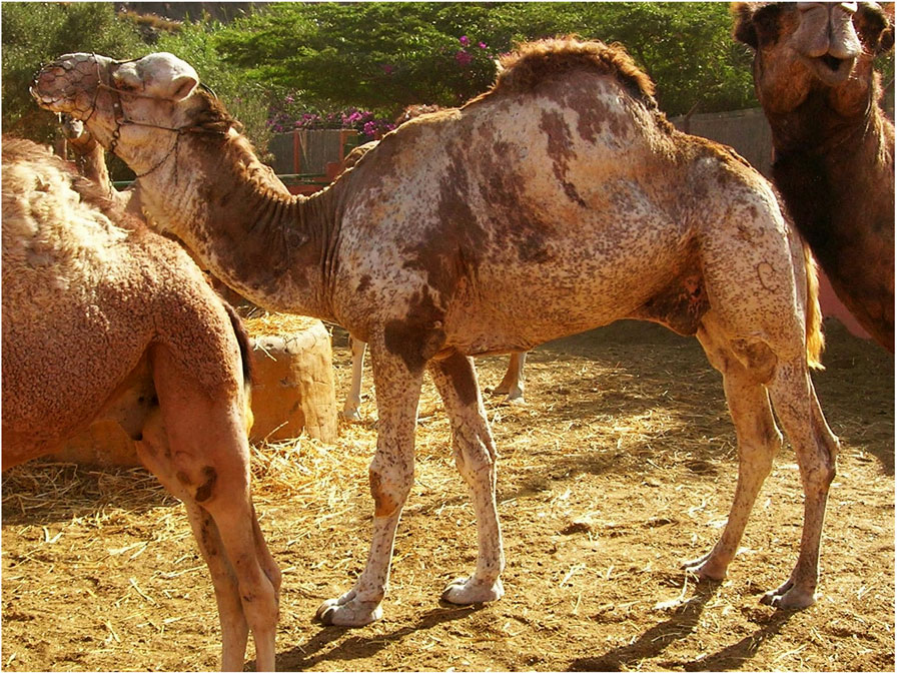
A camel bull of the Canary Islands showing a complex piebald pattern and speckled markings (Dioli 2013)

**Figure 4:**
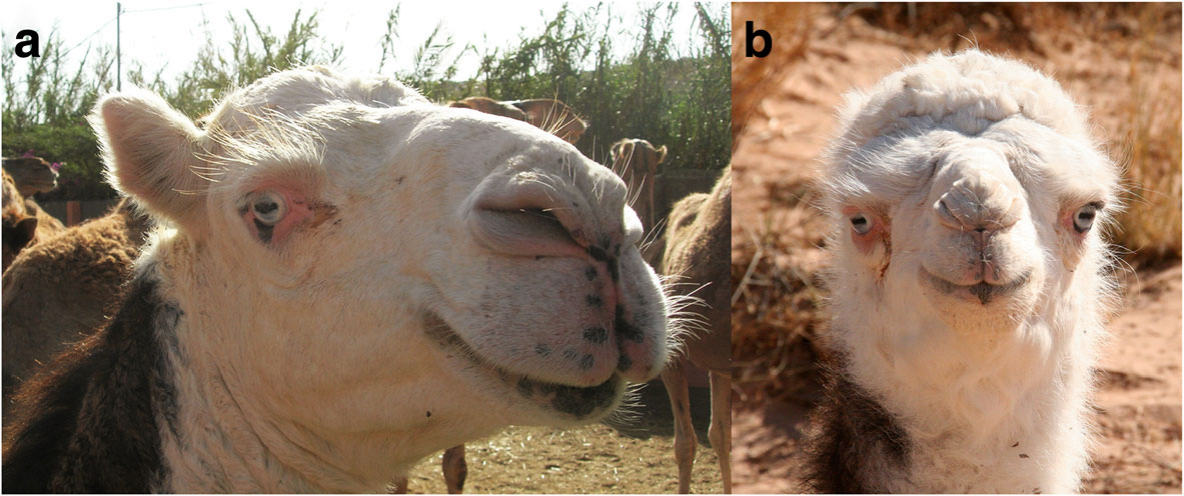
Heads of a juvenile camel (**a**) and of a calf (**b**) of Western Sahara showing leucism: pink skin and blue eye (GV, Dioli 2013)

**Figure 5:**
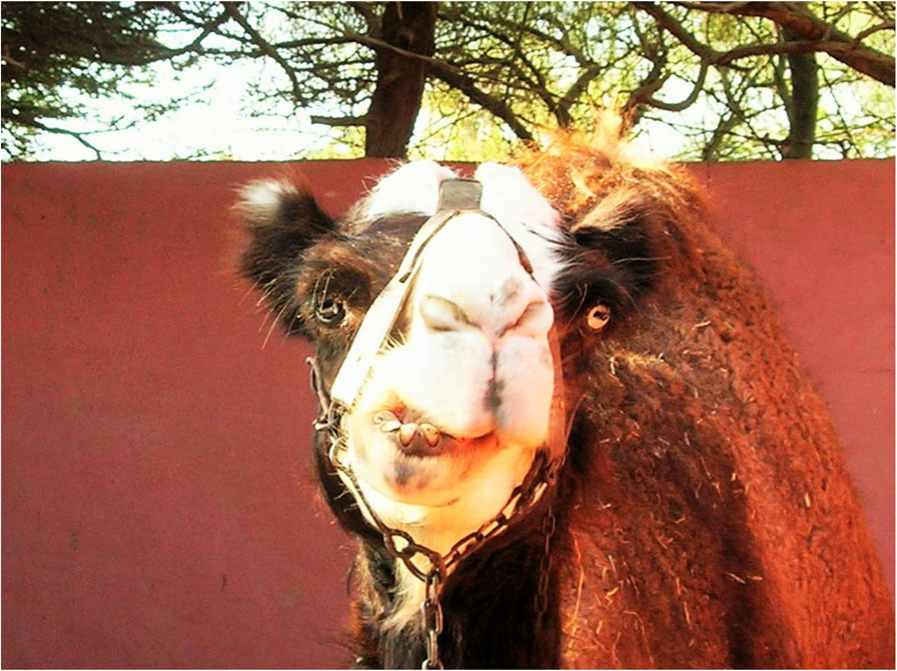
Head of an immature camel of the Canary Islands with eyes of different colours (Dioli 2013)

**Figure 6:**
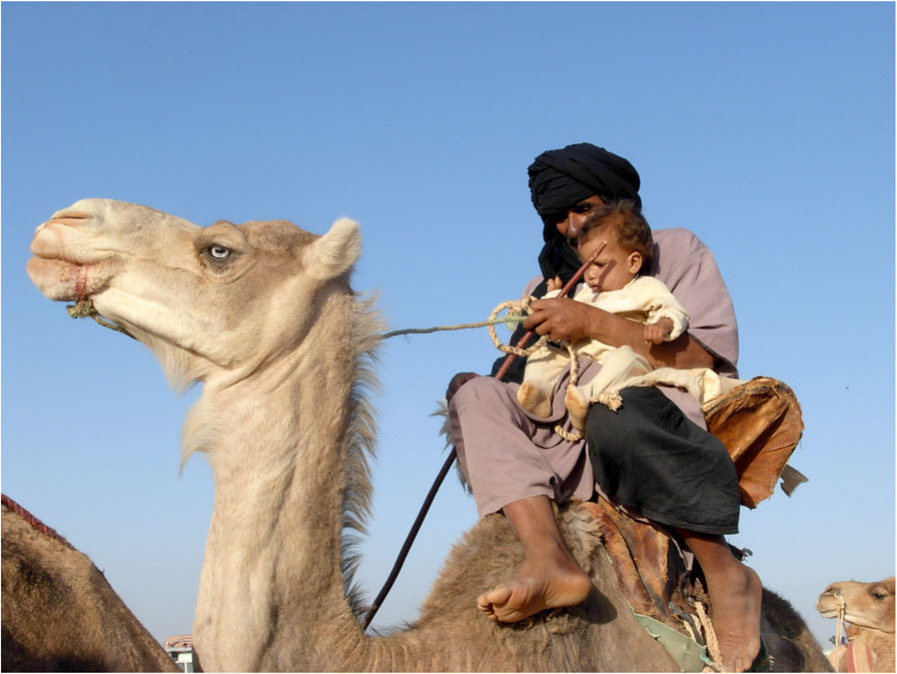
Riding camel of Western Sahara with solid coat and blue eyes (D. Rossi)

**Figure 7:**
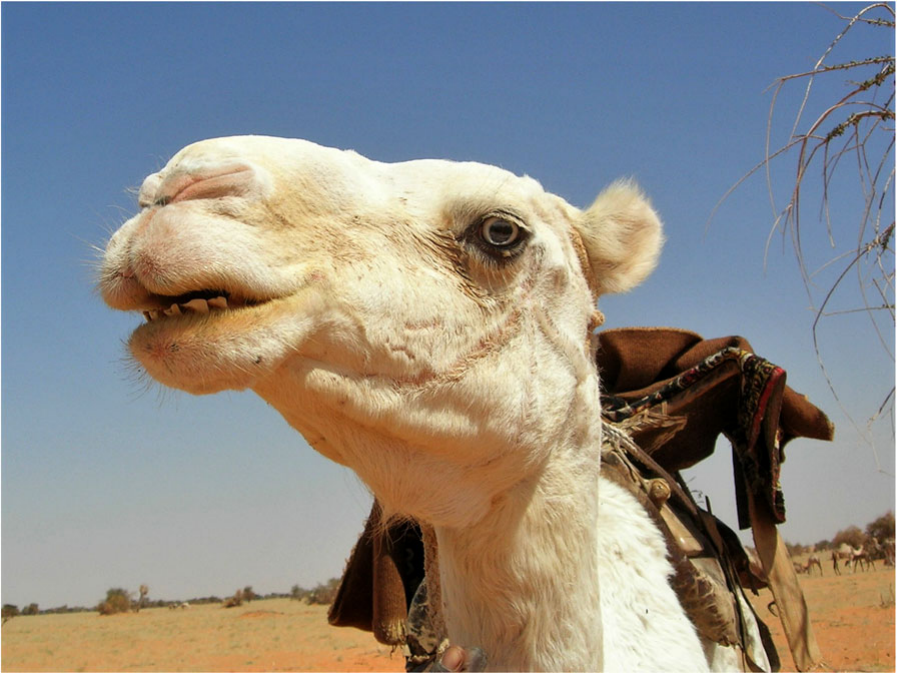
Male riding camel of Annafi breed from Eastern Sudan with solid coat and extremely pale iris (Dioli 2013)

Piebald camels have a white and solid (black, brown, tawny, red, or grey) coloration that varies between individuals in the relative cover of white or solid and in the shape that the patches assume on the body. There is a high variation in the amount of white in the body, from individuals who are all solid-coloured but the snout to others that are totally white or all white but the hump (Cauvet 1925; Mahaman 1979; Dioli 2013). This variation relates to the degree of leucism of the animal, which, with all evidence, has a genetic basis. From the available images of piebald camels (including those taken during fieldwork) and direct observations in the field, we can classify them according to the amount and distribution of white in the coat, clearly showing a characteristic progression of leucism among individuals, which starts from the snout and lower legs to progress commonly up on the head and on the belly and flanks (these seem to be the most common and shape-variegated camels), until covering all the body but the dorsal part (including the hump) (Figure 2). Camels displaying a high prevalence of white tend also to lose the contours of the solid-coloured patches, thus displaying speckled markings (Figure 3). Piebald camels with a prevalence of white on the head (i.e. most piebald camels) display blue eyes (Figure 4). Piebald camels may also have complete heterocromia of the iris, with one blue and the other brown in accordance with the coat colour surrounding the eyelids (Figure 5). In few cases, brown solid-coloured camels have blue or minnow eyes (Figures 6 and 7), and according to Sahrawi herders, this occurs in herds with piebald individuals. Toes may be white too, often in association with blue eyes.

Physiological characteristics include various degrees of deafness and visual impairments, and the notion that piebald camels with blue eyes may have audio and visual impairments is well known to African camel pastoralists (Faye et al. 2002). These impairments are the reason pastoralists regard piebald camels as reckless, stubborn, disobedient, or particularly tame, even numb. Indeed, a relation between depygmentation and similar variations in behaviour is well established in the literature for other domesticates. Sometimes, there are different levels of deafness and auditive impairments in affected animals - and apparently different predominant behaviours - in relation to the level of depygmentation: for example, Holstein cows with complete depigmented white areas on their heads are among the calmest, while those that are mostly white on the body are nervous and intractable (Grandin and Deesing 1998).^3^ Similarly, Sahrawi herders describe piebald camels as having different levels of deafness, with an increased deafness according to the presence of blue eyes and white colouring of the head and toes. Conversely, ‘when a boldpie camel has normal hearing, its black ears and nails are the sign of it’ (Monteil 1952). Perhaps complete deafness is related to calm behaviour, whereas partial deafness or hearing distortions are related to increasing agitated and unpredictable behaviour. Complete deafness may also be at the base of Sahrawi and Tuareg herders’ description of piebald camels’ behaviour as ‘stubborn’, ‘non-obedient’, and ‘hard in understanding orders’, but also as being quiet and tame and ‘following the rest of the herd’ (Migeon 2006). According to Tuareg herders, ‘camels with blue eyes are real nuisances. They suffer from bad eyesight at night and consequently often get lost when camel caravans move in the dark’ (Curdy 2001). Some piebald bulls are attributed a ‘low ability in managing the herd’ due to these impairments.

But piebald camels are also appreciated for their alleged courage in crossing barren deserts (Cauvet 1925), as well as for their alleged resistance to hunger, thirst, heat, and fatigue during caravan journeys. While it is well known that camels can drink brackish and salty water, Sahrawi nomads mention that piebalds can tolerate even higher concentrations of salts in drinking water. The belief that piebald camels are more resistant to thirst and heat may be associated with the increased albedo of white and light-colour hair upon exposure to the sun.

### Geography of piebald camels

**Figure 8:**
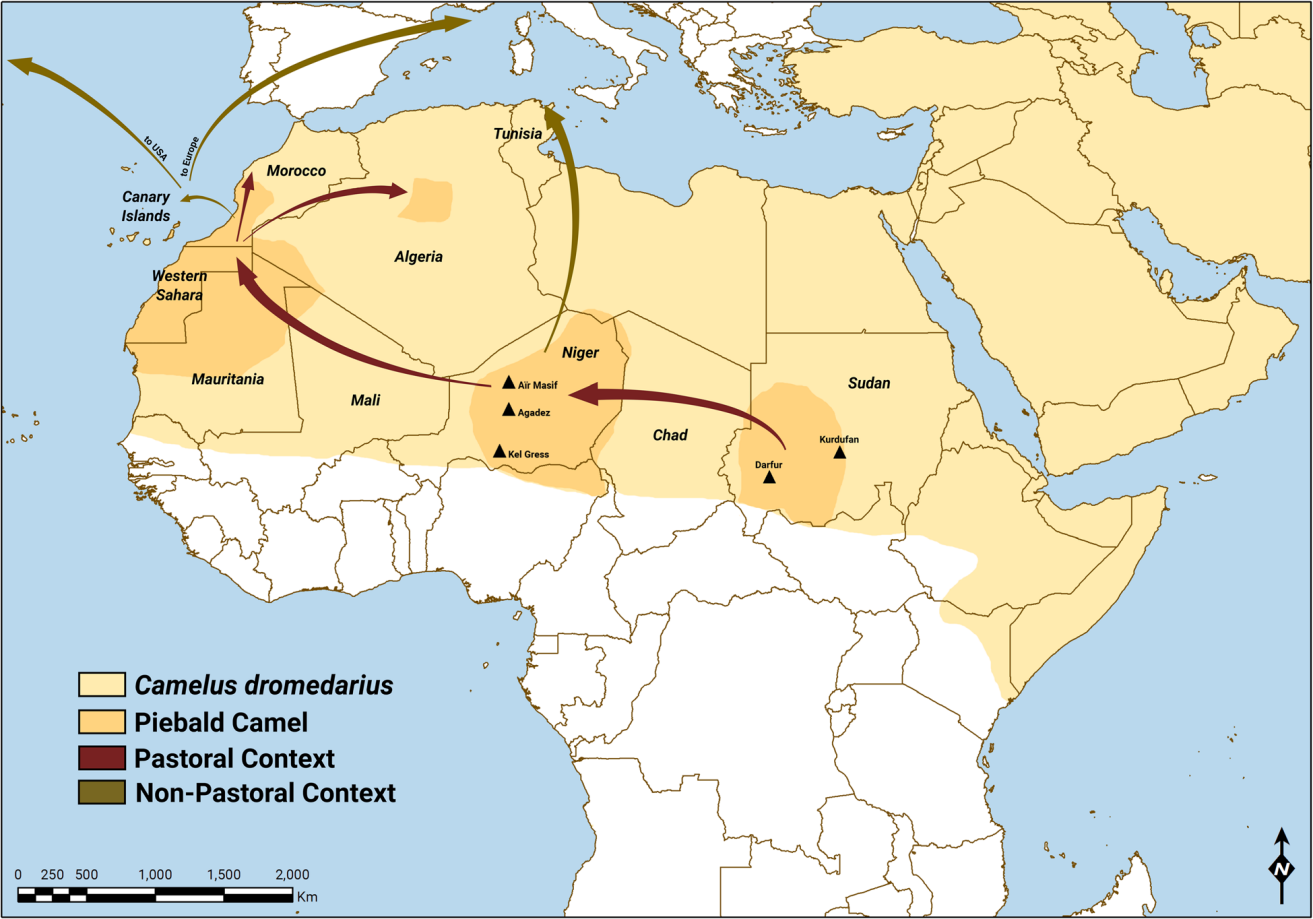
Areas of the world and names of the populations where contemporary breeding of piebald camels takes place (camel range is shown in *softer tone*)

Based on the available knowledge, the areas of the world and the names of the populations where contemporary breeding of piebald camels takes place are shown in Figure 8. Piebald camels are bred along a Southern Saharan fringe including Darfur (though in low numbers) and Kurdufan, Niger, Mauritania, Western Sahara, Morocco, central Algeria, and the Canary Islands. Few piebald camels are also present outside of Africa, namely in the USA and in Europe, where they have been introduced from the Canary Islands. We could not find any report, image, or direct observation of indigenous piebalds in the Middle East: there are no pied camels in Arabia (Faye et al. 2012) nor in Somalia (where the camel was introduced in earlier historical times from southern Arabia), the rest of the Horn of Africa, or among camel populations of the Mediterranean coast. Besides the few individuals exported to Europe, the USA, and few other areas, piebald camels live and likely originated in the Saharan Africa.

There is little information about the presence of piebald camels in the eastern Sahara desert. Leese (1927), cited in Köhler-Rollefson (1991), reports the breeding of camels with mouse-coloured backs and necks and white bellies, faces, and legs in Kordufan and Darfur. Wilson (1978) states that ‘occasional parti-coloured camels also occur’ in southern Darfur.

**Figure 9:**
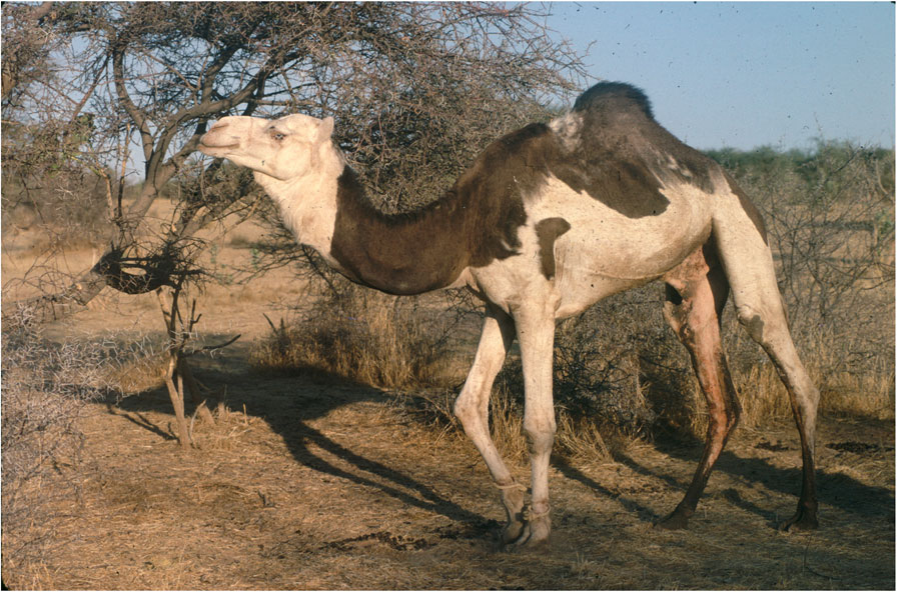
Piebald camel in Niger in 1994 (B. Faye)

Most of the information available in the literature about piebald camel breeding refers to the Tuareg of Niger (Figure 9). Some authors (Bernus 1969; CIRAD 2001; Pacholek et al. 2000a) report that the Kel Gress of central Niger are renown for breeding ‘spotted camels’, and the same is true for the Tuareg living in the region of Agadez (Chaibou and Faye 2003, Mahaman 1979; Antoine-Moussiaux et al. 2006; AA 2000). Others (Pacholek et al. 2000b; CIRAD 2001) list *azarghaf* (the local name for piebald camels) among the camel races/types bred in Niger and indicate that they originate in the south of the Aïr Massif. The presence of piebald camels in the region is reported also by Cauvet (1925), who describes a camel breed called ‘dromadaire de l’Azbin’ (Azbin is the name of the Aïr Massif in Hausa language) or ‘Haoussa de l’Aïr’ and reared by Tuareg tribes at the west and north of Agadez. Mahaman (1979), while classifying camel breeds of Niger, reports the existence of a breed called *azarghaf*, with minnow eyes and piebald coat, resistant and elected for long travels.

**Figure 10:**
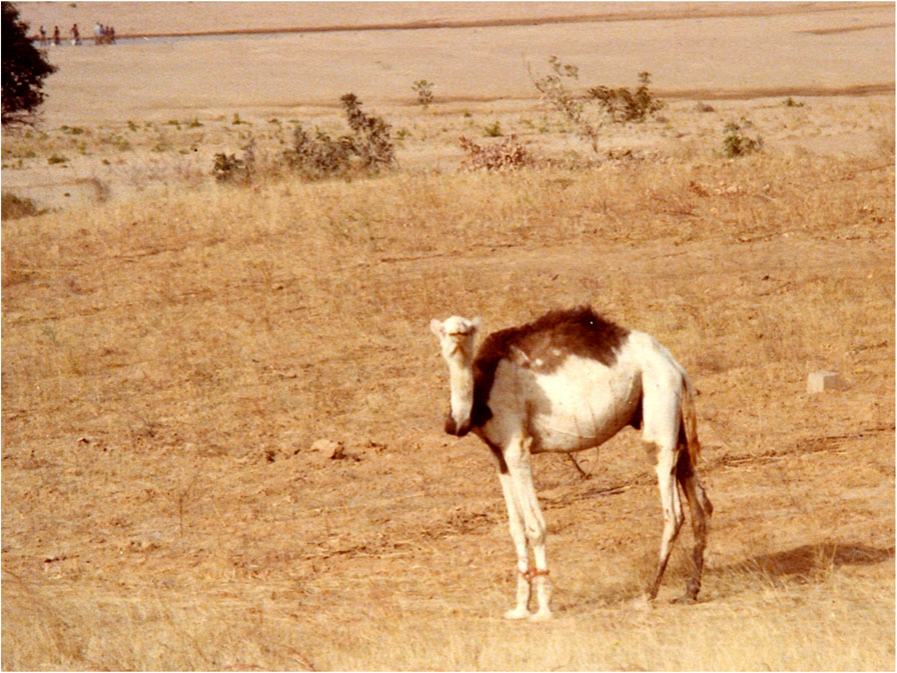
Piebald camel near the Bakolori dam in northwestern Nigeria in June 1978 (Giorgio Volpato)

Piebald camels are also known to and sometimes bred by Tuareg-neighbouring populations, mostly Hausa and Fulani of north Nigeria (e.g. in the Kano region), where the Tuareg Kel Gress move their herds during dry spells in Niger. This, for example, happened during the droughts of the 1970s: in Figure 10, a piebald camel near the Bakolori dam in Northwestern Nigeria in June 1978. These movements lie at the origin of the Nigerian pied camel breed reported in the FAO DAD-IS database.^4^

Besides the Tuareg, other pastoral populations renown for breeding piebald camels are the Hassanyia-speaking nomads of Mauritania and Western Sahara (Correra 2006; Monteil 1952), also known as Moors or, in the northwestern part, as Sahrawi. Among them, piebald breeding is an old practice that has lasted to the present day, as is the case for the Reguibat, the Oulad Delim, and the Oulad Tidrarin tribes (Mercer 1976; Caro Baroja 1955; Boyer 1962), as well as among Moorish tribes of present-day northern and coastal Mauritania (Correra 2006). Relatively small numbers of piebald camels are also bred by neighbouring (non-Hassanyia-speaking) pastoralists such as the Chaamba of Central Algeria (Shinar (2004), in citing Cauneille (1968), states that they have ‘dappled (patched) grey, brown, and dark copper’ coloured camels) and the Marazig pastoralists of South Tunisia, reported to have camels with a white snout (Boris 1951).

In the literature about camels in the Canary Islands, the origin, significance, and distribution of piebald camels are never addressed, although their existence among the herds of the islands is usually recognized. Camels were first introduced to the Canary Islands beginning with the fifteenth century by the Castilian conquerors; during the course of the following centuries, camels adapted to the islands’ climate and were used by local peasants, particularly of Lanzarote and Fuerteventura, to power agricultural implements (e.g. ploughs, watermills), as a riding and pack animal, and for wheeled transport (Morera 1991; Schulz et al. 2010). Piebald camels were further imported to the Canary Islands from Western Sahara (possibly from individuals originally belonging to the Oulad Tidrarin, as this tribe was customarily living along the coast of Western Sahara adjacent to the Canary Islands, with which it had historical commercial contacts). Indeed, a recent study has shown a clear genetic proximity between camel populations from the Canary Islands and from western Africa (Schulz et al. 2010), and Canary Islands’ piebald camels are reported as closely related to the piebald ‘Western Sahara’ breed (Dioli 2013), from which they likely originated. But when this happened is uncertain.

**Figure 11:**
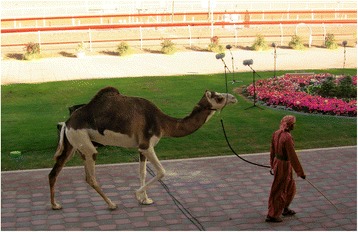
Piebald camel belonging to the Oman Royal Camel Corp (Dioli 2013)

Few isolated piebald camels live in zoological gardens, ranches, and farms outside Africa. Several private ranches and farms in the USA have piebald camels, descendants of piebalds that were shipped there from some place in Morocco or the Canary Islands a few decades ago. In Europe, at least two piebald camels are present in a herd of a Dutch farm producing camel milk for the European market,^5^ and some more live in a camping resort on the Mediterranean French coast, where they are used as amusement for children and for touristic parades;^6^ they all come from the Canary Islands. The Oman Royal Camel Corp (Royal Court Affairs) owns two piebald camels, likely imported from western Africa, and uses them for parades (Figure 11). In Tunisia, the Farhat Hached zoological park in Rades witnessed in 2008 the birth of a ‘blue-eyed dromedary of *mahri* breed with a rusty brown and white coat’. Media reported the news applauding at the birth of the ‘very rare species’, whose ‘immaculate blue eyes […] confer to the animal a singular and rare beauty’, with the calf bringing ‘great delight of the many children who visit the park with their parents’.^7^ This ‘very rare species’ is said to have been introduced to the park about two years before, to be usually raised by the Tuareg living on the border between Niger and Chad and ‘by Saharan tribes living between Mauritania and Morocco’.^8^

### Genetics of piebald camels

The piebald character is usually transmitted by one or more recessive genes that is (are) also responsible for blue eyes, congenital deafness, and impaired vision. Involvement of more genes and mutations make the genetics of leucistic animals complex (Householder 2003), and ‘the genetic pathways influencing coat coloration are still only poorly described’ (Cieslak et al. 2011). Unsurprisingly, given the lack of attention to leucism in camels in the scientific literature, no investigation has up to now been conducted on the genetics of piebald camels. From the information we collected, only a tentative hypothesis can be put forward, and in order to have a deeper understanding of piebald camels’ genetics, a ‘herd analysis exercise’ (Krätli 2008) among herds including piebald camels would be helpful.

In general terms, Sahrawi herders state that a piebald bull introduced into a non-piebald herd results in a 30% to 50% of piebald calves (males in most of the cases), that a piebald calf can be produced by two solid-coloured parents if there was a piebald in the family, and that conversely two piebald parents can produce solid offspring. According to the Sahrawi, piebald coloration and associated characteristics are transmitted to calves by blue-eyed piebald fathers. If the bull is not piebald, piebald calves are born in a relation of one third or one quarter to the number of piebald mothers. Hence, piebald condition in camels seems to be sex related, with males displaying the colouring pattern more often than females. Indeed, most of the piebald camels we saw during fieldwork in Western Sahara were males. Further insights can be obtained by comparing piebald camels’ genetics to that of other mammals, as it appears that there is a common genetic mechanism that determines coat colour (Wilkins et al. 2014). In Appaloosa horses, for example, the piebald expression is controlled by a single gene, which is apparently dominant, and displays the piebald pattern when two series of modifying genes are expressed: one series for the control of the expression of white (vis-à-vis the normal coat colour) and another series for the expression of spots (Householder 2003). Apparently, the expression of the white modifier and the spot genes are sex influenced, with heterozygote individuals expressing the characteristic when males, and not when females (Householder 2003), like in camels. In Dalmatian dogs, the piebald character is given by two recessive genes, with individuals displaying the phenotype when homozygous; the expression of the spotted colouring is farther related to the expression of genes for congenital deafness and blue eyes (Strain 1996), as seen in camels. The genetics of piebald camels may indeed involve not just one but more genes like in Appaloosa horses and Dalmatian dogs. Differently, in Holstein cattle, the piebald effect seems to be due to inheritance of a single piebald spotting gene, with no sex differentiation (Pape 1990), unlike in camels.

**Figure 12:**
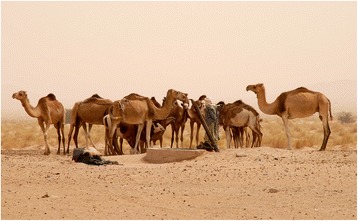
Camel herd at a Western Sahara well with roughly 30% of piebald camels (GV)

Being a condition transmitted by one (or more) recessive gene(s), leucism would appear in camel herds only at a very limited frequency in natural reproductive conditions, whereas the selection for leucism can be obtained in ‘close’ herds where the reproduction is selected for the appearance of recessive phenotypes. Herders favour piebald camels by selective breeding, but at the same time are aware of the risks of breed degradation and thus avoid inbreeding.^9^ As a result of the interplay between piebald genetics and selective breeding subjected to these two forces, the actual prevalence of piebald camels within a herd seems to be variable but seldom reaching the majority of the herds’ camels (Figure 12). This is indirectly supported by the analysis of camel herds’ composition in accordance to types/breeds around Agadez in Niger done by Chaibou (2005): in out of five camel breeds, the *azarghaf* (piebald) is one of the main breeds along with the *abzin* and *azawak*, and it is present in 55% of the herds; however, no one herd was composed only by *azarghaf* camels.

### Ethnotaxonomy

Throughout their range and among all the populations who breed them, piebald camels are known by names that recall their piebald coat or their blue eyes, less often their peculiar behavioural characteristics. Most names have an Arabic root.

Among Arabic-speaking populations (e.g. the Hassanyia-speaking Sahrawi and Moors and the Arabic-speaking Sudanese pastoralists), piebald camels are known as *azraq* (male), *zarqa* (female), or *zurq* (plural) (Taine-Cheikh 1989; Caro Baroja 1955). In classical and contemporary Arabic, *zurq* means ‘blue’, but also ‘blue-eyed’, ‘blue, minnow, or glittering eyes’, and ‘glaucoma’ (Fischer 1965). Though not in reference to piebald camels, the term has among some Maghreb populations a positive aesthetic connotation, with a female or woman called *zarqa* in reference to her sensual beauty (Roth 1985). In Hassanyia, the verbs *stazrag* and *zrāg* mean ‘being towards piebald coloration (of camels)’ and ‘becoming piebald’, respectively (Taine-Cheikh 2002), and *azraygat* their diminutive (Voisset 1989). The term *zurq* applied to coat colour is used also for a grey coat, especially in reference to horses and cattle (Roth 1985) but also in reference to camels, e.g. in Arabia (Abbas et al. 2000); nevertheless, to the Zaer of North Morocco, a *zurq* cattle is a cattle with white patches all over its body (Loubignac 1952). Interestingly, in colloquial Arabic there exists a derived term *mizra:qan*, used to indicate a camel delayed behind the rest of the caravan because it moves slowly (Allam 2000), which may be in relation with the tendency of some piebald camels to stray away. In Morocco and Western Sahara, the term *zeroual* is also used to indicate piebald camels, as well as people and camels with blue eyes.

There is a strong consistency in the terms used for piebald camels across time and space: they all originate from the Arabic *zurq*. The Tuareg use the *zurq*-derivated term *azarghaf*, not of Berber Tamasheq (Tuareg’s language) origin. This fact takes more significance when we consider that Tamasheq has a low proportion of Arabic loanwords, and usually few of these with regard to phytonymic and zoonymic terms: items lended/adopted from one population to the other are more likely to have the same etimology, and piebald camels and their zoonym may have been historically adopted by Tamasheq-speaking Tuareg from Arabic populations.

*Zurq* is also the etymological origin of some Spanish words, notably ‘*zarco*, −ca’ which means light blue, azure, and ‘*ojizarco*, −ca’ which means ‘blue-eyed’. Among Spanish-speaking piebald camels’ breeders of the Canary Islands, names for piebald camels also refer to their coat colour or to their impairments and associated behaviour. Piebalds are known as *manchado* or *pintado* (spotted or painted) when with white patches on the body or some of its parts and as *capiloto* when displaying a white head and a prevalently normal-coloured body (cfr. the Spanish *capirote*, i.e. cattle with the head and the body of different colours; Morera 1991). Among present-day Sahrawi of Western Sahara (a former Spanish colony), besides as *zurq*, piebald camels are also referred to as *colorín* (coloured) or *camellos zonzos* or *tontos* (silly, fool camels).

### Material and cultural importance

Piebald camels have different material and cultural roles among the distinct populations engaged in their breeding. These roles encompass subsistence material uses (e.g. for milk and meat production, transport, and caravans), a cultural importance in terms of aesthetics, cultural identity, and tribal/group identification, as well as the recent use of income generation out of a pastoral context.

In the Saharan areas where they are bred, piebald camels have been often used by different populations as pack camels for caravans, for transport (e.g. moving to new grazing areas), and as mounts because they are prevalently male and/or because of their resistance to thirst and heat and/or because their physiological impairments make them more tame. Among the nomads of Western Sahara and Mauritania, they were used in pre-colonial times as mounts by blacksmiths and griots as well as in caravans (Monteil 1952). They are used in caravans, particularly in areas with sandy soils (Migeon 2006), such as in Niger (Chaibou 2005; CIRAD 2001), where this breed is known ‘for its resistance and courage with which it crosses the desert from the Aïr to Bilma’ (Cauvet 1925). Indeed, they are used for the annual *tarlamt*, which are the salt caravans moving across the Ténéré desert from Agadez, Zinder, or Tahoua to the salt deposits of Fachi and Bilma in Niger (Cianchini 1999). In Morocco, they are used as mounts and to transport merchandise to towns (Driot 2009). In Nigeria, they are regarded as ‘very faithful beasts of burden used essentially for personal transportation, to carry loads, draw water, and pull ploughs’.^10^

In the Canary Islands, piebald camels are bred mainly to transport tourists (particularly in the island of Lanzarote), who seem to be particularly attracted to their variegated colouring and blue eyes (Dioli 2013). Nowadays, there are about 1,000 camels in the Canary Islands (Schulz et al. 2010), and they are almost completely used in the touristic industry. Many of them are piebalds. From the Canary Islands, piebald camels have been exported during the last decades to European and American countries (Castillo and Lugo 2003). In the USA, piebald camels are much appreciated aesthetically and valued two to three times the price of a solid-coloured camel (Berry 2006).^11^ The value of a piebald calf ranges between $3,000 and $6,000.^12^^,^ 13 Two farms in Tennessee hire their piebald bulls for breeding, while one farm in North Carolina, specialized in breeding ‘of rare and exotic animals’, sells its paint camels to private people or provide them for hire for tourist ‘safari’ (in ranches), commercials, theatre performances, and plays.^14^ Another ranch in Texas owns some fine piebalds (see pictures in the website)^15^; according to the statement in their website, they are breeding ‘for bigger and better spotted camels’.

Among Sahrawi and Tuareg breeders, piebalds are appreciated for their beauty in terms of variable patterns of coloration and blue eyes. They have become a marker of cultural identity and have been incorporated in poems, literature, folk knowledge, and foundation myths. They are considered a locus of visual stimulation and a source of positive emotions. Their high variability in coat colouring patterns have become an ownership mark with similar functions to those of customary livestock brands.

The positive selection for recessive genes for productive and/or aesthetic qualities is not new to livestock breeding. Some cultures regarded leucistic (piebald) as sacred animals, and they have even been the basis of legends and folklore: for example, Egyptian tomb paintings of 1400 to 1300 BC represent spotted horses (Householder 2003); ancient nomadic populations of Egypt and Sudan were known for appreciating and venerating piebald cattle, which was often represented in the rock art of the Central Sahara (d’Alverny 1950); and a type of piebald cattle was bred by the Zulu and regarded as royal cattle and held as almost sacred (Epstein 1955). The cultural identification of specific ethnic groups with clearly defined morphological characteristics (colour, size, horns, fattiness, etc.) of their livestock is a well-known although understudied fact in the history of human-livestock relations (Hunn 2011), and the relation of some pastoralists with piebald camels is an extraordinary case study. For example, in northern Nigeria, piebald camels are used as ceremonial animals during Salla Muslim celebrations.^16^ Piebald breeding can be achieved only by manipulating reproductive processes that favour the piebald character, and this genetic selection in turn becomes a cultural marker of piebald camel herders, i.e. piebald camels become means to construct and promote cultural identity, even as subjects of foundation myths. Here is a foundation myth passed down by members of the Oulad Tidrarin tribe of coastal Western Sahara (Caro Baroja 1955):

Sid Ahmed Bo Gambar, one holy ancestor of the Oulad Tidrarin, was one day praying on the beach, when suddenly an *azrag* [piebald] camel came out from the sea. Sid Ahmed took the camel, which was a stud, and from this camel the many *azrag* camels the Tidrarin owns descend

This myth attributes the origin of piebald camels to a miracle done by Sid Ahmed and explains the attachment and sense of identity that the members of the tribe have for these camels. In fact, nowadays, piebald camels are still a marker of cultural identity for the Sahrawi nomads (including the members of the former tribes of Western Sahara), who breed them particularly for their perceived beauty and the prestige derived from it.

The fact that leucism considerably varies the ratio of white to coloured hair between generations as well as between offspring from the same parents gives each individual a specific and recognizable pattern, which has often attracted pastoral populations (Rook et al. 1998). Sahrawi’s and Tuareg’s appreciation of piebald camels recalls the anthropology of aesthetics as discussed, for example, in the case of cattle among Nilotic populations (Coote 1994). Among the Mandari, a piebald ox is highly appreciated and, when a piebald is born, its owner is delighted and the beast is set aside for show (Buxton 1973). The high aesthetic value that the Mandari place on cattle patterning is explained by Buxton (1973) by the fact that, ‘they stand out strikingly in a landscape devoid of strong color’, where the individual beast provides the locus for stimulating visual experience (Coote 1994). Indeed, aesthetic explanations related to their striking and coloured appearance on an otherwise pale landscape are as appealing to camel-breeding nomads as they are for the cattle-breeding Mandari. Camels at the horizon are a common visual locus when travelling in Western Sahara. As piebald camels strike on the yellow horizon with a colour pattern variety, and as these coloured shapes vary from animal to animal, it is no wonder that a piebald camel represents a particularly appreciated visual stimulation.

Among the Tuareg, piebald camels are associated with beauty. To communicate their sense of beauty, Tuareg poets link man, camels, and pastoral resources. Piebald camels find a place in these poems. In one of them,^17^ the beauty of a girl is put in analogy with that of a piebald camel (Ghabdouane and Prasse 1990). Similarly, in a recent novel describing the commitment between a man and his piebald camel written by Al-Koni (2008), himself a Tuareg raised in the desert of Libya, a Tuareg nomad is constantly lauding his piebald *mahri* for the unique colouring of his pelt, in spite of its undisciplined and reckless behaviour. During a passage of the novel, a local sheikh entraps the beautiful piebald to get it to impregnate the sheikh’s she-camels and obtain calves from the piebald strain. In the sheikh’s words, it was ‘a piebald *mahri* as graceful as a gazelle. This line became extinct throughout the desert a hundred years ago’ (Al-Koni 2008). The blue eyes and coat colour and pattern of piebald camels have historically been at the centre of different emotional and rational attachments by their herders and are also much appreciated among tourists travelling to the Canary Islands and south of France, children visiting zoological parks (e.g. in Tunisia), and owners of rare and exotic animals in the USA.

### Are piebald camels a breed?

Wardeh (2004), in his classification of dromedary camels, does not refer to the existence of piebald breeds or individuals, whereas several other authors have described piebald camels as a camel breed/race (Pacholek et al. 2000b; Monteil 1952; Antoine-Moussiaux et al. 2007). From the information we collected, it seems that the piebald character may theoretically be present or introduced into different existing camel breeds, thus being a characteristic linked to different breeds (e.g. a piebald *mahri*) but it can also prevail over the traits of the existing non-piebald breed leading to the development of a new piebald breed. We can envision two ways through which a piebald camel breed originates: (1) A piebald camel (of a non-piebald existing breed) is born within a herd, and local herders, once it is sexually mature, favour its reproduction; (2) A piebald camel, preferably a bull, is introduced (e.g. through raid or purchase) into a herd with no piebald camels and selected for reproduction. By further selecting at the same time for the piebald character and for the characters of the original breed, the outcome in the long run will be piebald camels of the original non-piebald breed. However, protracted selection for the piebald character (rather than for the traits of the original breed) may create not a breed variant but new piebald breeds. In both cases, favouring the reproduction of piebald traits increases the pull of leucistic alleles in the herd (and then in local herds), so that in the long run, piebald camels are born with higher frequency. However, we have seen that piebald individuals are commonly a minority within herds due to the constraints of inbreeding and to the recessive character of leucism and these facts weight the balance toward considering ‘piebaldness’ a breed variant, maybe in the process of becoming a breed.

### Origin and diffusion of piebald camels

**Figure 13:**
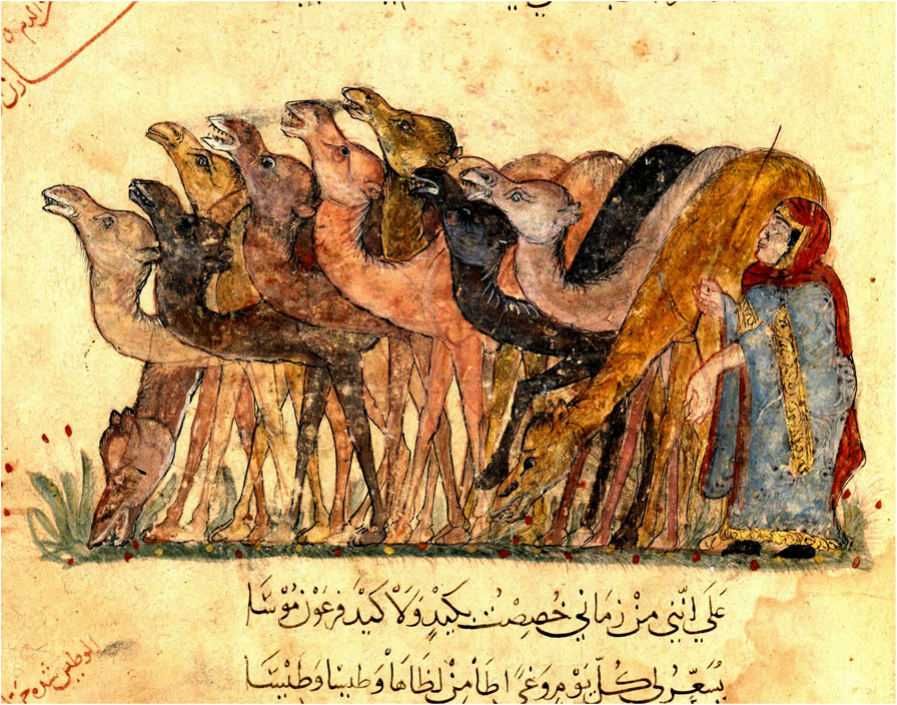
‘Herd of camels’, Arab miniature by Al-Wasiti from ‘Maqamat’ by Al Hariri, thirteenth century, Baghdad (Iraq), showing different camel coat colours. Note the absence of white camels, today common in many countries, and of piebald camels (courtesy BnF/National Library of France) (Dioli 2013)

When and from where did the breeding of piebald camels originate? Was it developed independently by different populations or spread throughout Africa from a single point and time of origin? We did not find any information about the existence of piebald Bactrian camels or of piebald dromedary camels in Asia, Australia, and the Middle East. Piebald camels are not represented in the plate showing camel coat colours in the Middle East and dated to the thirteenth century A.D. (Figure 13, from an Arab miniature by Al-Wasiti from ‘Maqamat’ by Al Hariri). The exclusive presence of piebald camels in Africa had been noted by Cauvet (1925), cited in Bulliet (1975), who considered piebald colouration as one among other distinctive characteristics that would prove that African and Asian dromedary camels are separate species and that the camel was domesticated in Africa independently from Arabian domestication. However, piebald colouration is more likely to be an indication of selective breeding for the piebald character once this has appeared by chance in domestic camels (Bulliet 1975). Historical trends of camel diffusion in the Saharan Africa and present distribution of piebald camels point to Kordufan or a neighbouring area as place of origin: some time after the introduction of camels in Northern Africa about 2,500 years ago, a piebald was born within one herd and this characteristic was readily picked up by local nomads. The resulting selection for piebald camels and their spread westward, and the appreciation of these camels by different Saharan pastoral populations, are likely to be at the base of present-day distribution of piebald camels throughout Africa.

When and where domestication took place is still controversial for many species, and Cieslak et al. (2011) suggest that ‘at least in some cases, coat and skin color can be a valuable marker for resolving these discussions.’ Although the question of camel domestication has been complicated and frustrating for many scholars, there is some agreement that the camel, after having been hunted between 6000 and 3500 BC, has become the focus of direct domestication in the third millennium BC probably in south Arabia (Clutton-Brock 1988; Bulliet 1975, Zeder 2012). Sherratt (1983) suggests that camel domestication took place in two developing zones of long-distance trade (Bactrian camel toward the east, dromedary camel towards the south-west) in relation with the growing urban area of Mesopotamia. The camel as a pack and military animal was at its greatest expansion during the Nabatean times (second century BC - first century AD), during which period camels would have been taken up by populations in the eastern Egyptian desert (e.g. the Beja).

Camels had been replacing cattle and horses throughout the southern edge of the Sahara from approximately the third century BC, and a variety of populations of camel nomads have subsequently developed from Sudan to Mauritania and Western Sahara, through Niger, Mali, and Algeria. There is some agreement among scholars that camels spread westward through Africa first from a south-Saharan route going from Sudan to Mauritania about 2,200 years ago and shortly later through a north-Saharan route, reaching western Sahara from both routes about 2,000 years ago (Blanc and Ennesser 1989; Wilson 1984; Bulliet 1975). The diffusion of camel husbandry and nomadism facilitated a growth of a trans-Saharan caravan trade in the north-south and east-west directions. Indeed, the trading route going from Sudan and the Nubian desert to Timbuktu in Mali, and passing through Darfur, Kano, lake Chad, and Gao, was active and established already in 1400 (Wolf 1982).

The camel nomads of southern Sahara developed their own distinct forms of livelihoods and culture (e.g. in uses of camels and camel products, saddle technologies, taste, and aesthetic preferences), which included a preference for piebald camels. This preference, combined with the trans-Saharan exchange networks, facilitated the diffusion of piebald camels. Indeed, ancient Egypt and Sudanese nomadic populations were known for appreciating and venerating piebald cattle (d’Alverny 1950), and a breeding preference for piebald livestock (e.g. cattle, sheep) was already established in the region before the camel’s introduction. This preference may have combined with a genetic drift occurring in the relatively low number of camels supported by the Saharan environment and taking part in this westward dissemination. This genetic drift may have caused the appearance of otherwise relatively rare conditions such as leucism and the associated piebald traits. This may at the same time explain the lack of piebald camels in Arabia and Somalia, where camels were present in huge numbers since the dawn of camel domestication and where leucism would have been quickly damped out by the much broader gene pool. All this suggests that the pioneer piebald camel breeders were relatively few, their livestock herds small in size, and their ability to control breeding well developed (Bulliet, pers. comm.).

From western Sudan, piebald camels would have spread (through raids, exchanges, and purchases) westward and their breeding being taken up by the southern Tuareg first and by Moorish populations of Western Sahara and Mauritania then (Figure 8). For example, according to Mahaman (1979), ‘[*azarghaf* camels] can be held by other ethnic groups who purchase them from the Tuaregs to use them essentially as pack animals.’ This supports the hypothesis that piebald camels of Western Sahara find their origin from purchases at the markets of Niger, as expressed by older living members of the Reguibat tribe: the first piebald camel - they say - was a bull brought from the east about four centuries ago (between the sixteenth and the seventeenth centuries).

The paths of camel diffusion support the idea that similar paths have been used by piebald camels in their diffusion and the contemporary distribution of piebald camels supports the idea that they spread through a south-Saharan route. Through this route, piebald camels were adopted and bred by populations who had already shown an appreciation for other piebald livestock. During the following centuries, piebald camels spread through the Sahara from east to west of Africa, and further into the Canary Islands, Europe, and the USA. Overall, the history of piebald camels have much in common with the history of other piebald breeds around the world, as, for example, the one of splashed white Icelandic horses.^18^ Splashed white blue-eyed horses of Iceland are the outcome of a genetic drift that isolated the population coupled with a recent upsurge in an aesthetical preference for these traits among Icelandic horse breeders.

### Trends in piebald camels today

Based on the total number of camels known to occur in piebald breeding areas, on piebald genetics, and on direct observations, we roughly estimate that there may be less than 5,000 piebald camels in the world. Most are in Niger and Western Sahara, bred by the Tuareg and the Sahrawi, respectively. In different areas, piebald camels are subjected to distinct trends affecting their numbers and material and cultural importance.

At least three different major contemporary trends can be identified as involving piebald camels: (1) their decline due to the abandonment of nomadic livelihoods (due to droughts, wars, destocking, etc.) and due to camel commodification and market pressures against piebald camels; (2) their disengagement from a pastoral economy and their use as source of income in the touristic and entertainment industries due to their aesthetical qualities; and (3) a resurgence of piebald camel breeding as a marker of political and cultural identities.

The first trend seems to be taking place in Sudan due to expansion of crop farms, droughts, and wars (Musa et al. 2006), as well as in Niger due to market pressures. In both areas, the demise of caravans in favour of motorized transport during the last century has surely contributed to a progressive reduction of piebald camel husbandry, the more so considering their widespread use in caravans. In Niger, there seems to be an ongoing process of further disengagement by many Tuareg herders. According to Pacholek et al. (2000a), piebald camels in the south of the Aïr are threatened by ‘absorption’ by the other two non-piebald local camel breeds, the *azawak* and the *manga*. This is attributed to the fact that ‘the monetarization of pastoral societies pushes herders to turn to the breeding of types with the highest market value’, and as piebald camels are less valued (e.g. for milk and meat production) on local markets, herders introduce non-piebald bulls into their herds (Pacholek et al. 2000b).

The second trend is evident in the Canary Islands and the USA, where piebald camels are actively bred and used in the touristic and recreational industries. Ranches in the USA have started raising piebald camels during the last two decades: they are appreciated aesthetically; they have higher monetary value than solid-coloured camels and are actively sold or hired to a variety of actors (Berry 2006), thus further spreading in new areas of the world, albeit out of a pastoral context.

**Figure 14:**
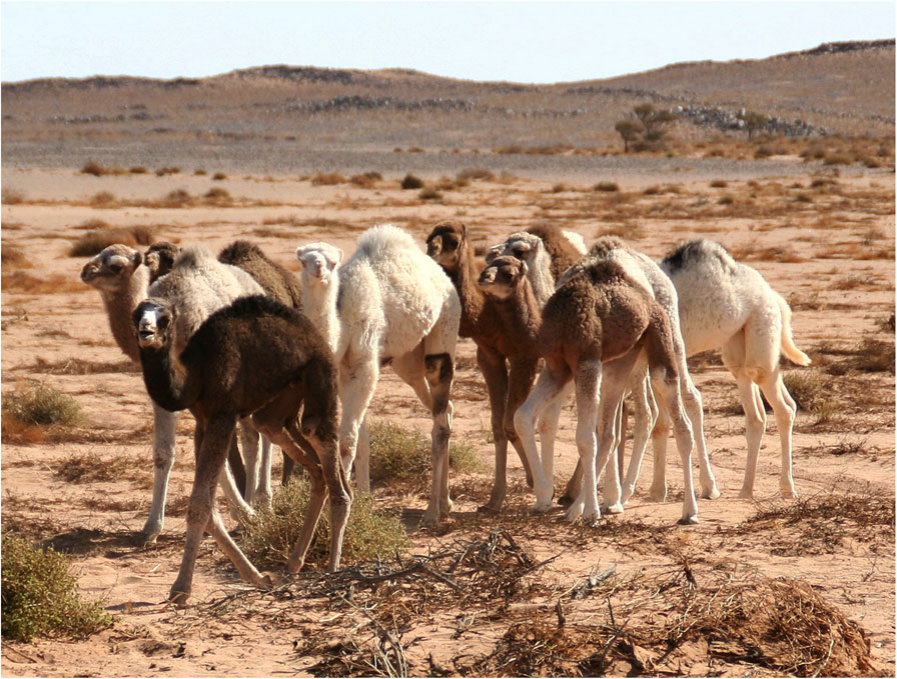
A group of piebald calves in Western Sahara (A. Broglia)

The Sahrawi exemplify the third process: during the twentieth century, with colonialism, droughts, and then the Morocco-Polisario war over Western Sahara (1975 to 1991), piebald camels (and nomads) confronted a drastic reduction in numbers. During the war, camels were killed and herds bombed and others were abandoned in the desert by their fleeing owners and either died of hunger or thirst or were used by soldiers for meat provision. In the refugee camps where the Sahrawi ended up living, camels were absent and the social entities that used them as objects of cultural representation (i.e. tribes, fraction) destroyed overnight. But starting with the 1990s, Sahrawi refugees regained access to camels and camel husbandry, to grazing territories, and to traditional camel-related knowledge, and re-established relations with local ecologies (Volpato and Howard 2014; Volpato and Puri 2014; Volpato et al. 2013). Sahrawi refugees and nomads recovered the breeding of piebald camels for the same material and cultural reasons but with a new cultural referent: the Sahrawi people. Piebald camels became a marker of Sahrawi identity and a statement of cultural and political freedom, besides being appreciated for their aesthetical qualities. Piebald camels are a fairly common encounter nowadays in Western Sahara, particularly in its inland part under Sahrawi-Polisario control (Figure 14).

Although camel breeds have been historically important in the ability of pastoral populations to adapt and better exploit the desert environment, with increasing commoditization of camels and camel products, there is the risk that some locally adapted camel breeds may be diminishing in population and slowly disappearing (Blanc and Ennesser 1989). With the possibility that negative trends may prevail in different areas of Africa, it is urgent to study piebald camels and cultures around them. In reference to Nigerien piebald camels, studies report that it is undergoing a ‘progressive disappearing of a type [of camel] with marked characteristics…and maybe unique…This danger deserves to be taken in consideration in regard to the safeguard of…biodiversity’ (Pacholek et al. 2000b), as well as cultural diversity.

## Conclusions

This study has addressed the history, geography, and anthropology of piebald camels. Based on fieldwork among the Sahrawi of Western Sahara, direct observations across north and east Africa and the Middle East, and a literature review, the case illustrates how piebald camels are an integral part of the story of the camel and of camel pastoralists in Africa and how piebald breeds are an interesting case to understand the dynamic relations of pastoralists with their livestock. Piebald camels probably originated in the eastern Sahara hundreds of years ago from a mutation that was picked up and selected for by local pastoralists who already had a preference for piebald livestock. In the ensuing centuries, piebald camels spread westward throughout Africa while being adopted by distinct pastoral tribes and groups across present-day Sudan, Niger, Mali, Mauritania, Western Sahara, and Morocco. They were bred for their alleged docility, for resistance to heat and thirst, and for their aesthetical value and were used in caravans and as mounts, as well as for subsistence purposes (e.g. milk and meat production) and for cultural reasons (e.g. as marker of cultural identity). During the last decades, piebald camels have been exported to the Canary Islands, Europe, and the USA, where they are used out of a pastoral context in the touristic and entertainment industries due to their perceived beauty and tameness.

The findings of this study are significant for understanding trends related to breeds and breeders in contexts of historical and contemporary change in pastoral systems. The findings also contribute to providing insights into the more general process of human selection for piebald colorations in domestic animals. This process can have different drivers and motives in different socio-ecological contexts and time. Piebald camels are under threat due to wars and droughts that kill herds and undermine pastoral livelihoods, e.g. in Darfur. They are threatened by increasing commodification of camels and camel products and consequent herd homogenization towards breeds with high marketing value, e.g. in Niger. Piebald camels and their husbandry have seen a resurgence during the last two decades as a symbol of political and cultural identity, in Western Sahara. Further studies are needed to fully elucidate the biology and history of piebald camels and their roles and dynamics in the present and in historical time. Fieldwork in other geographical areas (other than Western Sahara) where piebald camels are bred would be of great help in this sense. Also, a genetic study may help to identify different strains and to reconstruct piebald camels’ temporal and geographical evolution and diffusion. A research into old written sources, mainly of Arabic origin, could help clarify the timing when piebald camels came to the Saharan Africa. Further studies would also be interesting to understand the place of piebald camels as an aesthetic *locus* among desert pastoralists. Such studies could make an interesting contribution to the field of anthropology of aesthetics.

We have pointed out that, in distinct breeding areas, piebald camels are subjected to negative trends, which increase the possibility of losing important genetic traits as well as the associated biocultural diversity (e.g. genetic, knowledge, and management). If these negative trends prevail, we risk losing the results of centuries-old engagement of humans in piebalds’ breeding. The full story of piebald camels may be lost before having the chance to be told.

## Footnotes

1 A *mahri* or *mehari* is a Sahelian riding camel

2 It is important to clarify the distinction between piebald animals whose white areas are depigmented (e.g., Dalmatian dogs, Overo and Tobiano horses), and those that have a white coat color but are fully pigmented (e.g., Arab horse, Brahman cattle) (Granding and Deesing 1998); only the former are leucistic animals.

3 Holstein cows with blue eyes are nowadays selected against and excluded from inclusion in the European Union register of accepted breeds.

4
http://www.fao.org/dad-is/

5
www.kamelenmelk.nl

6
http://www.marina-plage.com/v2/

7
http://www.letemps.com.tn/article.php?ID_art=16296

8
http://allafrica.com/stories/200805121059.html

9 Inbreeding this kind of animals might cause neurological abnormalities, as it has been shown for dogs and cats (Grandin and Deesing 1998).

10
http://www.fao.org/dad-is/

11
www.camellosafari.com

12
http://www.camelphotos.com/cgi-bin/ads.cgi?ct=baby_dromedary&md=second&id=286&min=&max

13
http://www.camelphotos.com/cgi-bin/ads.cgi?ct=baby_dromedary&md=second&id=243&min=&max

14
http://www.ferncroftfarms.com/Home_Page.html

15
http://www.hiviewfarms.com/images/exotics2.htm#Camels

16
http://www.fao.org/dad-is/

17 Ezzara, tu es plus belle que les jeunes chamelles au milieu desquelles se dresse un étalon brun Celui aux taches noires sur la croupe et la tête, ce chameau pie aux (aisselles) blanches…’

18
http://icelandmonitor.mbl.is/news/nature_and_travel/2016/06/12/the_incredible_blue_eyed_horses_of_iceland/
